# Detecting Rising Wildfire Risks for South East England

**DOI:** 10.1002/cli2.70002

**Published:** 2025-01-09

**Authors:** Vikki Thompson, Dann Mitchell, Nathanael Melia, Hannah Bloomfield, Nick Dunstone, Gillian Kay

**Affiliations:** ^1^ School of Geographical Sciences University of Bristol Bristol UK; ^2^ Royal Netherlands Meteorological Institute (KNMI) De Bilt Netherlands; ^3^ Climate Prescience Limited Rotorua New Zealand; ^4^ Climate Change Research Institute Victoria University of Wellington Wellington New Zealand; ^5^ School of Engineering University of Newcastle Newcastle‐upon‐Tyne UK; ^6^ Met Office Hadley Centre Met Office Exeter UK

**Keywords:** climate change, large ensemble climate models, UK, Wildfire

## Abstract

In July 2022 southeast England experienced a record breaking heatwave and unprecedented wildfires in urban areas. We investigate fire weather trends since 1960 in southeast England using a large ensemble of initialised climate models. Record smashing temperatures coincided with widespread fires in London, and we find that while wildfire risk was high, it was not record breaking. We show that between the 1960s and 2010s annual maximum daily fire weather has increased. The proportion of summertime days with high and very high fire risk has increased—while medium and low risk days have become less common. These findings show the need to mitigate against the increasing risk of wildfire caused by climate change.

## Graphical abstract

In July 2022 southeast England experienced a record breaking heatwave and unprecedented wildfires in urban areas. We investigate fire weather trends since 1960 in southeast England using a large ensemble of initialised climate models. We show that between the 1960's and 2010's annual maximum daily fire weather has increased, with a halving of the return period of a high risk day from a 1‐in‐4 year event in the 1960s to a 1‐in‐2 year event in the last decade.

Modelled change in daily fire weather risk for South East England. Showing the percentage change between the past (1961–1970) and present (2013–2022) of total summertime days within each fire danger class, using the large ensemble of initialised climate model data.

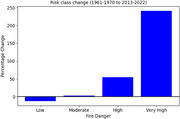

## 1 Introduction

In this warming world wildfires are a growing risk to society and ecosystems (Jones et al. 2022; van Oldenborgh et al. 2021). Though not entirely controlled by meteorological conditions, the weather does play a role in wildfire risk. Other factors, such as land use and societal behaviours, also affect the risk (Brown et al. 2016). Fire weather is the weather conditions conducive to the ignition and spread of wildfires. Several meteorological factors play a role—higher temperatures, longer dry periods, and greater winds increase the chance of wildfire, while precipitation and low wind speeds dampen the risk (Bedia et al. 2015; Richardson et al. 2022). We are experiencing increasing temperatures, magnified heatwaves, and intensified droughts across the globe (IPCC 2023), increasing the chance of fire weather in summer.

There are many ways in which wildfire impacts society. Wildfires destroy property, infrastructure, and agricultural lands (Kovats and Brisley 2021). Wildfire impacts human health over a range of timescales (Finlay et al. 2012). As well as direct risk of injury or death there is a risk of smoke inhalation and mental health impacts—with both immediate and long‐term effects (Caamano‐Isorna et al. 2011; Graham et al. 2020). Wildfires lead to long‐term water and soil pollution, impacting ecosystem health (Finlay et al. 2012).

**FIGURE 1:**
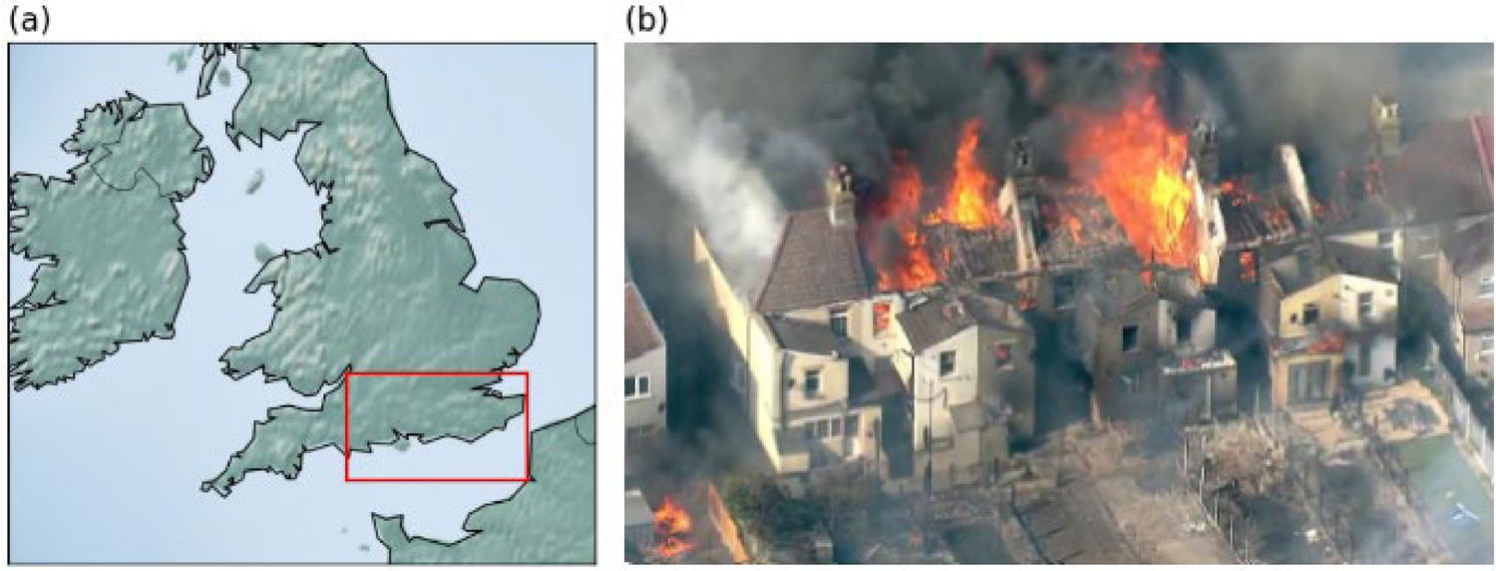
London wildfires of 2022. (a) Showing the region of southeast England (50.2 to 51.7°N, 1.5 to –2.5°W) used in this study, only the land area is included. (b) The impacts of urban wildfires in London in July 2021 (photo: Sky News).

In England, wildfire risk is small compared to many other global regions (Arnell, Freeman, and Gazzard 2021a; Arnell et al. 2021b; Perry et al. 2022). Despite this, impacts from wildfires have already been notable. In south England most wildfires occur in the peak of summer (Nikonovas, Doerr, and Santin 2022)—often linked with periods of extreme heat. In July 2022 the United Kingdom exceeded 40°C for the first time, with 40.3°C recorded in Coningsby, Lincolnshire (Yule et al. 2023). On July 19, at the peak of the heatwave, numerous wildfires broke out across England including many in urban areas in southeast England. Such fires in urban areas can lead to greater impacts due the surprise element—in London these events are unexpected and thus not prepared for. London fire brigade declared a major incident due to the number of fires, their busiest days since World War II (Arnell 2022). The wildfires destroyed 41 properties in London, with extensive transport infrastructure damage and air quality reduction due to smoke (Figure 1). As most English wildfires are ignited, usually unintentionally, by humans, behavioural changes can reduce risk. With better understanding of the changing risks caused by climate factors local authorities can use policy changes and education to limit impacts.

Wildfire has not previously been perceived as a major risk in the United Kingdom, thus there are few UK specific studies. In England and Wales fewer than half of the Community Risk Registers mention fire, though awareness is increasing (Arnell, Freeman, and Gazzard 2021a). Recent trends indicate that burnt area and wildfire numbers in the United Kingdom are increasing (Belcher et al. 2021), though recent trends in burnt area are not statistically significant, and there is a high level of interannual variability (Nikonovas, Doerr, and Santin 2022; Perry et al. 2022). The UK Climate Change Risk Assessment (CCRA3) Evidence Report 2021 notes that the more serious recent wildfire in the United Kingdom have occurred on heath or moorland, and most studies focus on these habitats (e.g., Glaves et al. 2020). CCRA3 considers a major wildfire spreading into residential areas as high impact, low likelihood (Kovats and Brisley 2021). The 2018 UK Climate Projections (UKCP18) have been analysed to investigate projected future fire weather changes (Arnell, Freeman, and Gazzard 2021a; Belcher et al. 2021; Perry et al. 2022). Perry et al. (2022) find up to 50% of summer days with high fire danger for London at 4°C global warming. However, there is a lack of studies quantifying the present day risk which can put the extreme event of 2022 into a long‐term context.

We assess fire risk in southeast England in summertime. Different regions experience peaks in wildfire activity at different times—even within the United Kingdom. The peak in south England is summertime, when at least double the number of fires are detected compared to other seasons (Nikonovas, Doerr, and Santin 2022).

In this study we assess southeast England fire weather using the Canadian Forest Fire Weather Index (FWI). This was developed in 1968, and has been used operationally since 1971 (Turner and Lawson 1978). It assesses the wildfire risk based on meteorological conditions. This index does not assess the chance of a wildfire occurring, but the intensity and rate of spread once started—the fire potential. Although first designed for Canadian forests, FWI has been applied to many regions globally and shown to be a useful measure (Abatzoglou, Williams, and Barbero 2019; Krikken et al. 2021). For example, it has been used in New Zealand (Melia et al. 2022) and Portugal (Carvalho et al. 2008). Some studies do make alterations to allow for science development and regional differences such as daylight hours (Arnell, Freeman, and Gazzard 2021a), but these would not affect long‐term trends.

Attribution studies of wildfire events have been conducted previously. The 2017 extreme fire season in Canada led to an assessment of components of fire weather—temperature and precipitation individually—finding temperature has increased significantly likely leading to the higher burnt areas observed (Kirchmeier‐Young et al. 2019). Significant wildfires in Sweden in 2018 led to a study assessing the influence of climate change on the FWI, finding that the short observational record did not allow for detection of trends (Krikken et al. 2021). FWI has also been used to attribute the 2019–2020 Australian bushfires, identifying trends in observations and some models, finding some drivers do show an imprint of human‐induced climate change (van Oldenborgh et al. 2021). These studies highlight the complexity of detecting trends and attributing wildfire due to the multiple drivers. Some studies attempt to overcome this by assessing only one driver, such as temperature (Brown et al. 2023). In this study we assess the risk of fire weather—other studies have used measures such as burnt area (Krikken et al. 2021) or fire growth (Brown et al. 2023).

Here, we use a large ensemble of initialised climate models to evaluate trends in wildfire in southeast England since 1960. Using a large ensemble, rather than only observations, enables trends to be identified sooner than if we waited for confidence in real world observations alone. We have up to 20 ensemble members for each calendar year—rather than a single year available from real world observations—allowing the emergence of statistically significant trends from a shorter time period. We begin by investigating the model fidelity in the climatic variables used to calculate the FWI, to ensure the climate model can be used as a proxy for observational data. We then use the large ensemble of climate model data to assess the chances of extreme fire weather conditions. Finally, we assess the trend in the FWI between the 1960s and the 2010s.

## 2 Methods

### 2.1 Data

To calculate the FWI four daily climatic variables are required: noon values for temperature, relative humidity, and wind, and the total precipitation for the preceding 24 h (noon to noon). As these exact variables are not available for the model we instead use maximum daily temperature, daily mean relative humidity, wind speed, and total daily precipitation. The difference between the required variables and those we use leads to a slight underestimation in FWI (Figure S1).

We use the fifth generation ECMWF atmospheric reanalysis of the global climate (ERA5) dataset as a proxy for observations (Hersbach et al. 2020). This reanalysis datasets provide spatially complete gridded climate data by combining observational records with data from forecasting models and data assimilation systems used to fill gaps where direct observations are unavailable or unreliable. Although over the region assessed there is good availability of direct observations, using ERA5 allows the climatic variables required to all be obtained from the same dataset rather than multiple data sources which may have differing spatial or temporal resolutions, or be physically inconsistent with each other. The daily maximum temperature and wind speed, and total daily precipitation are derived from the hourly outputs. Relative humidity is calculated from daily mean temperature and dewpoint, using the following formula (Alduchov and Eskridge 1996):

$$\text{RH}=100\times \left[\frac{e^{\frac{17.625\;\times \;D_{p}}{243.04\;+\;D_{p}}}}{e^{\frac{17.625\;\times \;T}{243.04\;+\;T}}}\right],$$

where RH is relative humidity, *D_p_* is dewpoint temperature, and *T* is temperature.

Climate model data from the hindcast ensemble of the Met Office Decadal Prediction System version 4 (DePreSys4) are used (Hermanson et al. 2023; Kay et al. 2023). The system uses the Met Office global coupled climate model HadGEM3‐GC3. The model has an atmospheric resolution of 60 km in the midlatitudes, and an ocean resolution of 0.25°. The hindcast ensemble has 10 members initialised each year from 1960 to 2020, on November 1st. For this study, for each ensemble member the first two years of data are used, providing a total of 1220 (61 years × 10 members × 2 years) summer seasons, spanning 1961 to 2022. The earliest model month evaluated is the 8th simulated month—beyond the deterministic range of near‐term model prediction. The daily maximum temperature, maximum wind speed, relative humidity, and total precipitation are model outputs.

For both the reanalysis and model southeast England, 50.2 to 51.7°N, 1.5°W to 2.5°E, is extracted, and area averages calculated with the sea masked (Figure 1). The region is based on the England south‐east and central south climatological district defined by the UK Met Office. It includes much of the impacts seen in July 2022—and particularly the surprising urban wildfires within London.

### 2.2 Calculating the Fire Weather Index

FWI is calculated using the original Canadian Forest Fire Weather Index (Turner and Lawson 1978; Wang, Anderson, and Suddaby 2015; Figure S2). Adaptations can be made to adjust for geographical variability and improvements in understanding over time (as in Arnell, Freeman, and Gazzard 2021a) but for this study we choose to make no changes. As we are comparing model to reanalysis data, providing the same calculation method is used for both it is a fair comparison with no need for local adjustments.

FWI is calculated iteratively, and along with the four climatic inputs several intermediate indices from the previous day are required, leading to the calculation of a daily FWI. It is calculated for every day annually, but we evaluate only summertime (June, July, and August).

### 2.3 Assessing Model Fidelity

**FIGURE 2:**
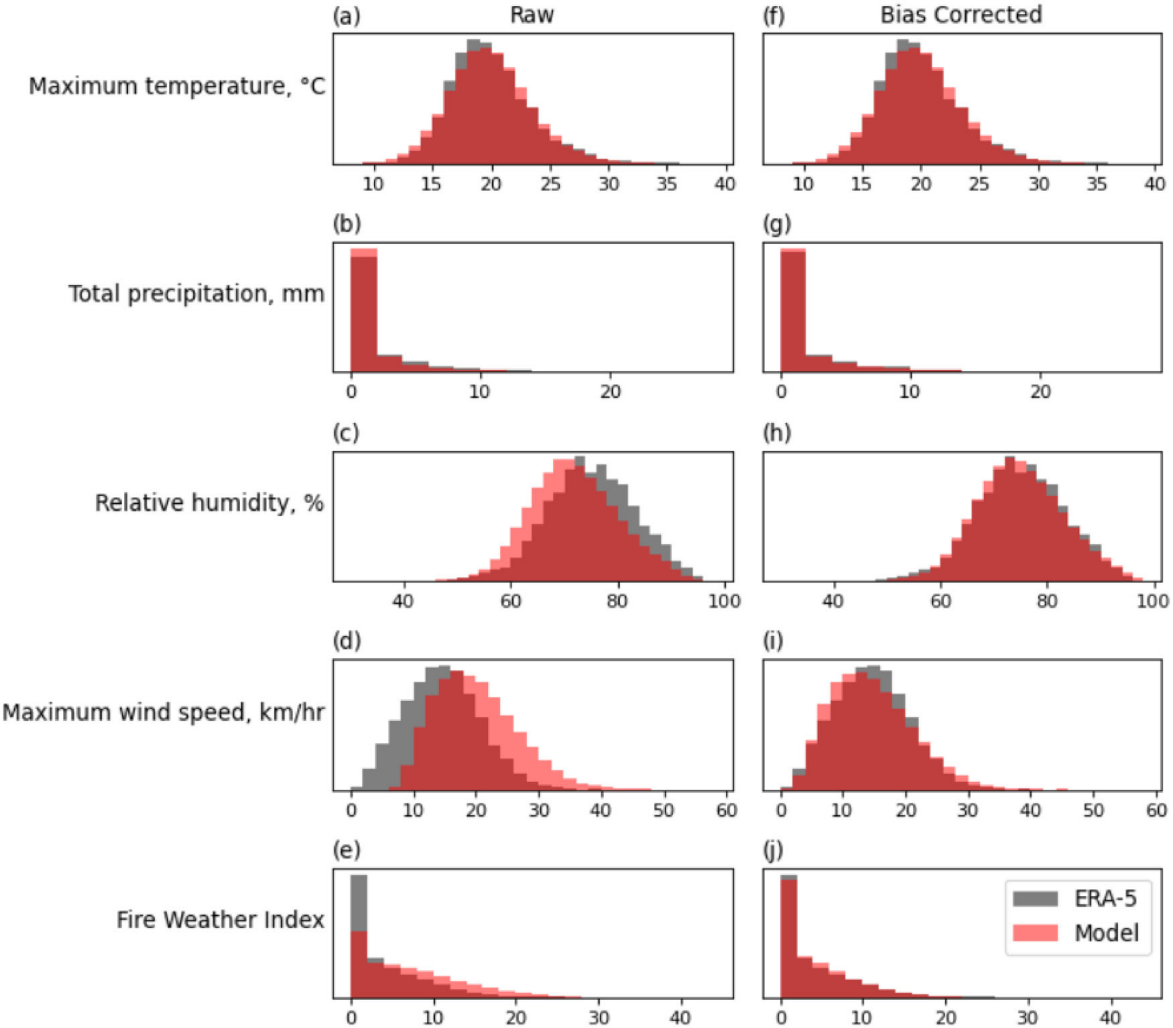
The distribution of the meteorological input variables and resulting fire weather index. (a–d) The ERA5 reanalysis (grey) and DePreSys4 model (red) distributions of 1960 to 2022 June–July–August daily maximum temperature, total precipitation, relative humidity, and maximum wind speed. (e) ERA5 reanalysis (grey) and DePreSys4 model (red) distributions of fire weather index calculated from the values in (a–d). (f–i) as in (a–d) but with DePreSys4 model distributions adjusted to the reanalysis data (see Section 2). (j) Fire weather index distributions recalculated from the adjusted model values.

We assess if the model climatology of each variable is consistent with the reanalysis, and make corrections as required. The distributions for the variables are shown in Figure 2. Without any model bias correction, the modelled and reanalysis distributions show considerable offset for wind, and some offset for relative humidity. This does not necessarily mean the model is doing poorly—there is evidence of biases in the reanalysis. Possible biases in the reanalysis wind data have been previously identified, with ERA5 showing lower summer winds over Europe than HadISD observations (Molina, Gutiérrez, and Sánchez 2021).

The offsets in input variables cause an offset in calculated FWI, which would prevent any reliability in the modelled fire weather extremes. The model variables are adjusted to better agree with the reanalysis, with FWI recalculated from the corrected model variables. We correct only bias in the mean, as if we adjust the model data too much we remove the advantage of using a large model ensemble as extreme value analysis of observations alone would provide the same results. We assess the relationship between the variables before and after the correction to ensure that it is not greatly changed (Figure S3). FWI distributions for the first and second summer in the climate model were compared and found to be indistinguishable. Therefore all climate model data can be considered together with one bias correction applied. For consistency, a correction is applied to all 4 of the variables, for temperature, relative humidity, and wind speed the model data is shifted by the difference between observed and model mean, ensuring the character of model variability is preserved. For precipitation linear scaling by a multiplicative adjustment, based on the difference between the observed and model means, is used to prevent the possible inclusion of negative precipitation (Lenderink, Buishand, and Van Deursen 2007; Zhu et al. 2022).

As we have corrected the model data to fit the reanalysis data the absolute values of FWI are comparable—but may differ from what would be found if we used a different dataset. For assessing changes in the chance of exceeding thresholds this is not an issue as we are comparing like for like. We could not compare the model FWI values calculated from other datasets, such as those from Met Office fire severity maps, or compare to different observational and reanalysis datasets without further bias assessment.

### 2.4 Assessing Risks and Trends

Using the model data as a large ensemble of physically plausible event days we can calculate the chance of any individual day exceeding various thresholds (Thompson et al. 2017). The annual daily maximum FWI of 2022 and 1976 are chosen as two notably extreme years. The full dataset from 1961 to 2022 is used, but if there is an increasing trend in the data this method will lead to an underestimation of the risk in the present day.

We can assess the changing risk of wildfire using fire danger classes (Perry et al. 2022; Table 1). Counting summertime days within each fire danger category for the model and the reanalysis we assess how the fire danger has changed between the past (1961–1970) and present (2013–2022). We show the uncertainty range in the model ensemble by calculating the proportion of days in each category for the individual ensemble members and including this range (Figure S4).

**TABLE 1:** Fire danger classes.

FWI	Range
Low	< 4.54
Moderate	4.54–9.38
High	9.38–17.35
Very high	17.35–52.36
Exceptional	> 52.36

*Note*: Each day can be classified into fire danger classes based on the fire weather index (FWI). The categories are defined by the UK Met Office (Perry et al. 2022).

To investigate trends we compare the annual maxima for the past and present, using the first and last decade of the model data. We use extreme value theory to calculate risk curves for each decade (Coles 2001). A generalised extreme value distribution was fitted to the data and used to calculate the return levels. Uncertainties are calculated by applying parametric bootstrapping 1000 times and taking the 2.5%–97.5% range (Kyselý 2008). We also calculate the return period for a high risk day occurring in a given year, for each year and each decade, to ensure we are identifying a trend and not sampling different phases of decadal variability (Figure 6a).

## 3 Results

The four climatic variables used as inputs to calculate FWI are shown in Figure 2. ERA5 reanalysis data is taken as a proxy of observations, and model data is a large ensemble of initialised climate models, DePreSys4 (see Section 2). The large ensemble provides ∼20 times more data than is available from observations, potentially allowing more extreme, rarer, conditions to be sampled.

**FIGURE 3:**
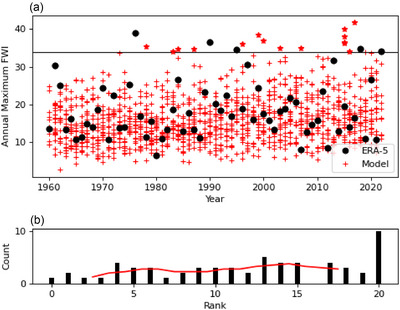
Is the model a fair proxy? (a) Reanalysis annual maximum values (black) and modelled annual maximum values for each ensemble member (red). The black horizontal line indicated the fire weather index 2022 observed maxima. (b) The rank of the reanalysis within the 20 model ensemble members for each year (A rank of 0 indicates the reanalysis value is lower than any ensemble member, 20 indicates the reanalysis value is higher). The red curve indicates a 5‐point running‐mean, to help illustrate the (lack of) trend.

We validate the model‐derived FWI by applying a fidelity test (Figure 3). The variance and trend of the model and reanalysis is compared, using methods from Suarez‐Gutierrez, Milinski, and Maher (2021). We take the annual summertime maximum FWI for each ensemble member and rank the observed annual maximum within the ensembles (Figure 3a). The ranks for each year are shown on a histogram (Figure 3b). Zero indicates the observed value is lower than any model ensemble member and 20 indicates the observed value is higher than any ensemble member. If the model has greater variability than the reanalysis the ranks would be more likely in the middle of the range, if the model and reanalysis trend disagree the ranks would be skewed. We find the model may underestimate the extremes, as the reanalysis shows a bias towards a rank of 20. This suggests we will be underestimating risk when assessing using the modelled FWI values.

**FIGURE 4:**
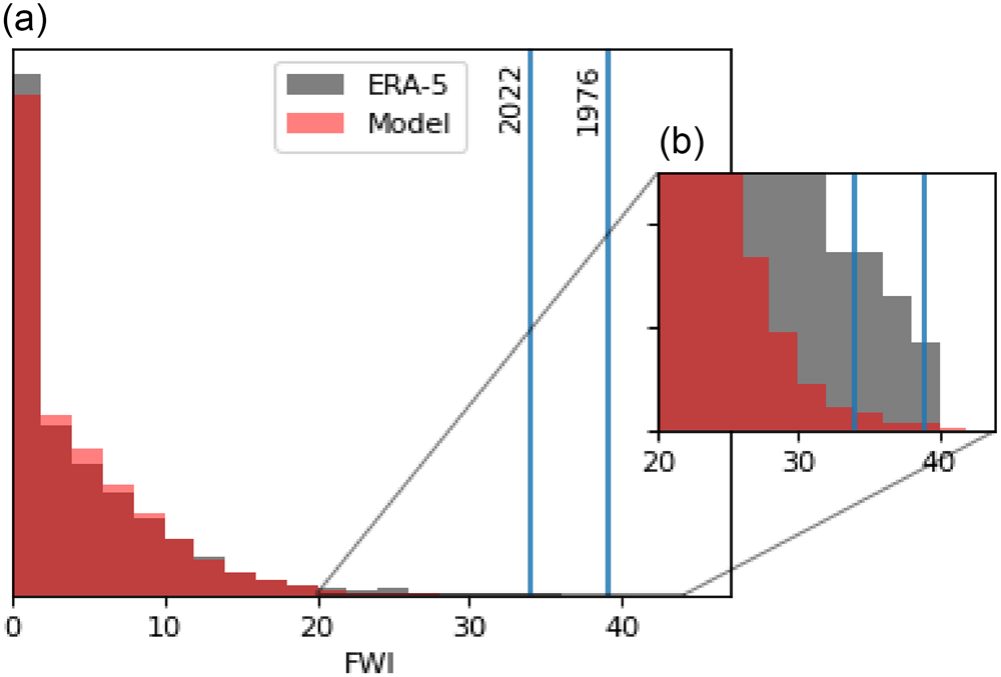
Chance of extreme fire weather index. (a) The distribution of ERA5 reanalysis (grey) and adjusted DePreSys4 model (red) JJA daily fire weather index, with vertical lines at the 1976 (right) and 2022 (left) annual maxima for the reanalysis data. (b) The upper part of (a) magnified to highlight the reanalysis and model days above the thresholds.

Comparing the reanalysis and model distributions for FWI we find several unprecedented FWI in the model ensemble (Figures 3a and 4). The record day in 2022 is July 19, but it is only the 5th highest annual maxima in the series. The greatest FWI is in 1976, a year known for its unusually dry and hot summer across the United Kingdom (Kendon et al. 2024).

Using annual maximum JJA FWI from the model ensemble over the full period, the exceedance likelihood for key events can be calculated. We assess the exceedance likelihood for the July 2022 event and the 1976 maxima. These estimations are likely to be conservative, as any climate change trend is likely to be increasing FWI values. We find the chance of exceeding the 2022 maxima in any given year is 1.4%, and the chance of exceeding the higher 1976 maxima is 0.2%.

**FIGURE 5:**
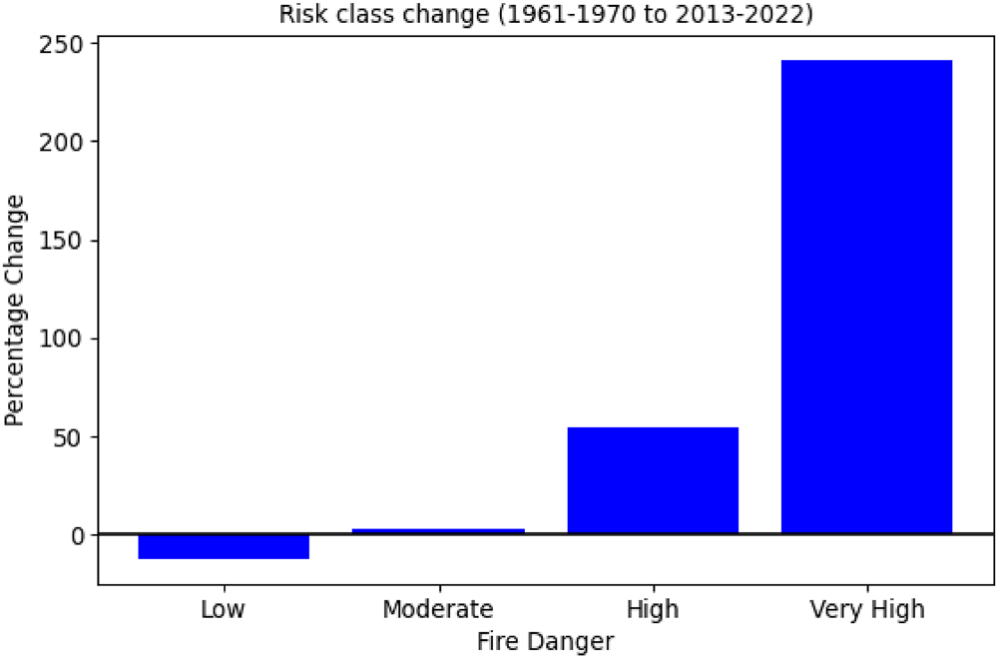
Modelled change in daily fire weather risk. Showing the percentage change between the past (1961–1970) and present (2013–2022) of total summertime days within each fire danger category (Table 1, n.b. no exceptional risk days occur), using the large ensemble of initialised climate model data.

The large ensemble allows us to calculate the exceedance likelihood thresholds within a single decade with greater certainty, as we have ∼20× more data than using reanalysis alone. For two individual decades—1961–1970 and 2013–2022—we assess the chance of each summertime day falling within various fire danger categories, low to exceptional (Table 1; Perry et al. 2022). We find no days in the exceptional class, in agreement with Perry et al. (2022). We calculate the proportion of days within each risk class for model and reanalysis (Figure S3). The reanalysis values lie within the range of individual ensemble members for all risk levels. Comparing the proportion of days in each risk level in the two decades the change in risk can be assessed (Figure 5). Since the 1960s, there is a shift towards higher risk levels. The number of days in the low risk class has fallen. Very high risk days have increased by 240% and high risk by 55%. It is noted that although the number of high risk days is shown to more than double these days only make a small proportion of all summer days. Most days remain in the low risk class.

**FIGURE 6:**
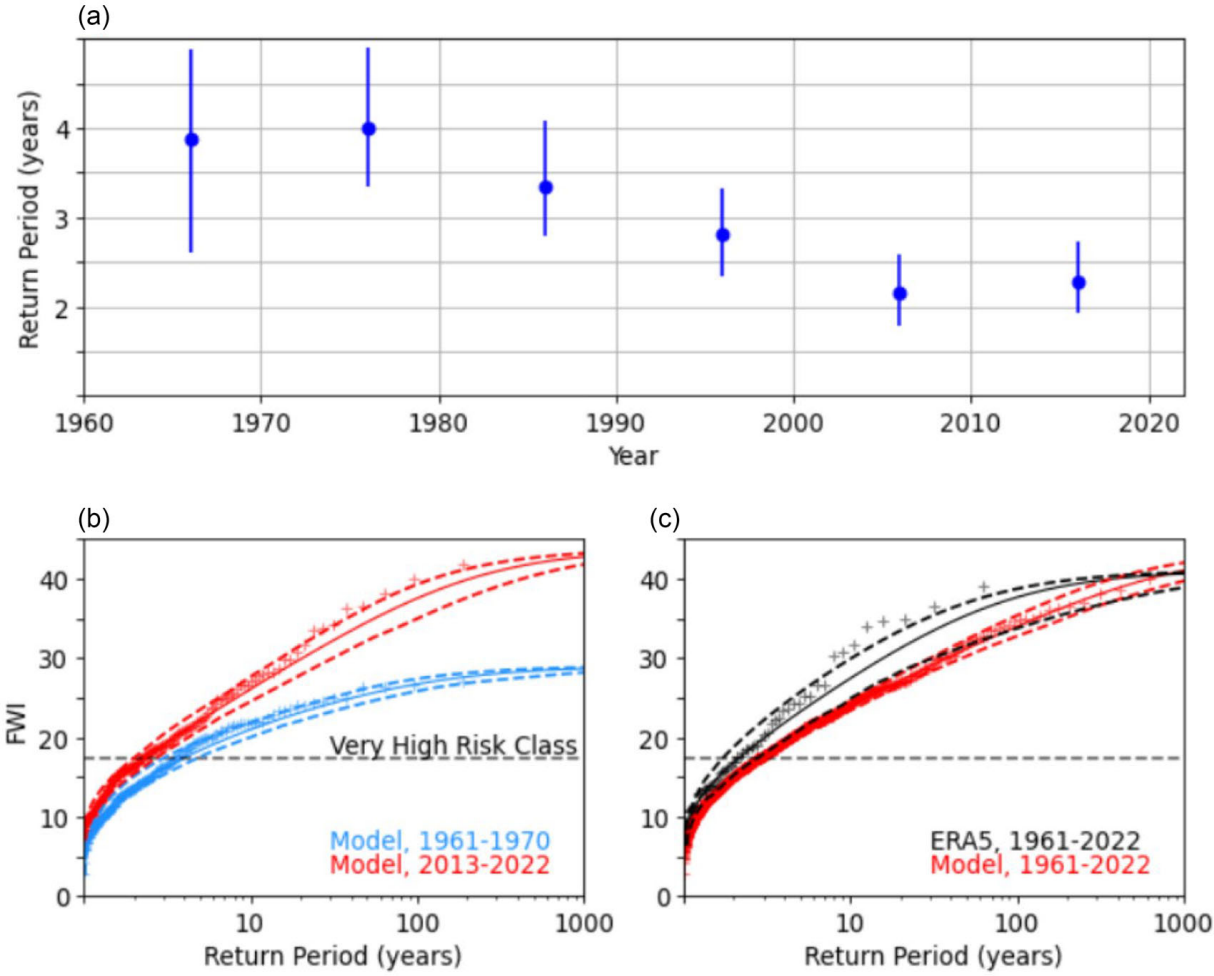
Detecting change in annual maximum fire weather index. (a) Return period of the annual record being a high risk day (FWI > 17.35) for each decade, with the 95% certainty range indicated. (b) Return period curve for the model data from 1961–1970 (blue) and 2013–2022 (red) annual maximum fire weather index. Crosses indicate the model points, solid line a generalised extreme value estimate with dashed lines indicating the 95% certainty range. Dashed horizontal line at FWI of 17.35, the lower limit of the very high risk class. (c) As in (b), but for the full dataset, 1961–2022, for the model (red) and the reanalysis data (black).

The initialised model large ensemble allows trend assessment with greater reliability than from only reanalysis. We use the model data to calculate return periods of FWI for each year and decade (see Section 2, Figure 6a). Between the 1960s and 2010s the return period has decreased from 3.9 (2.6 to 4.9) years to 2.3 (1.9 to 2.7) years. We show the return curves for annual maximum FWI for the first and last decade (Figure 6b). Figure 6c shows the advantage of using a large ensemble over reanalysis alone. The large ensemble provides ∼20× more data, enabling greater constraint of the return period. The reanalysis curve lies above the model, showing the model slightly underestimates the risk. With only 10 years of reanalysis data available per decade it is not possible to compare the return period curve of the first and last decade of data.

## 4 Discussion

Despite the numerous observed wildfire events in southeast England in July 2022 it was not unprecedented in terms of FWI, within the ERA5 reanalysis. In urban regions, localised microclimates may have further increased FWI, leading to greater FWI than is observed over the region as a whole. The FWI does not include all meteorological factors that affect fire risk—for example low amounts of cloud cover on July 19 would have acted to increase ignition likelihood (Vinokurova, Solovyeva, and Filippova 2022).

Many social factors will have increased the risk of wildfires in the United Kingdom on the hottest day. For example, lack of air conditioning may have led to more people seeking cooler conditions outside. An increase in outdoors social gatherings, including BBQs and people smoking, would lead to an increase in unintentional wildfire triggers. The risks of wildfire in urban areas can be reduced through warnings and legal enforcement, as is the case in other regions such as Spain. In urban regions where wildfire is closely related to social influence the FWI may fall short—temperature alone likely becomes of greater importance.

In this study, only FWI is assessed. There are other variables that could be used, for example Fine Fuel Moisture Content (FFMC). FFMC estimates the moisture content of surface litter and fine fuels and is particularly responsive to relative humidity and temperature changes. It represents the soil surface layer conditions and thus may be more suitable in wetter regions—it has been shown to be a good representative of fire risk in Ireland (Met Éiraenn 2024). For more drought prone regions, such as southeast England, it does not perform as well. Different components of the fire weather system (Figure S1) peak in different seasons, but the FWI peaks in summertime for England, therefore is suited to this assessment. Indeed, the UK Met Office fire warning system is based on FWI; values are translated to Fire Severity Index, as in Table 1, and these are used to trigger fire prevention actions for England and Wales (UK Met Office 2023). If applying the methods to other regions consideration of the best metric would be needed. Other metrics which could be more applicable in different regions include Vapour Pressure Deficit or Hot Dry Windy index (Srock et al. 2018).

The shift from lower fire danger days to higher will lead to a change in behaviour of fires that are ignited (Turner and Lawson 1978). Fire weather does not control the ignition of fires, but those fires that do occur on lower fire risk days will smoulder and are unlikely to spread, thus they will be easier to extinguish. In contrast, at higher fire danger any fires that are ignited are more likely to spread rapidly and burn more intensely—requiring greater resources and faster responses. Understanding the risks of such occurrences is important to fire brigades for planning purposes.

We only evaluate the summer season, and only in one region of the United Kingdom. The season and region were chosen due to the impacts observed in summer 2022. Similar assessments could be carried out for other regions to help understand whether trends are driven by climate change, or other causes such as land‐use changes. For example, in recent years the Scottish Highlands have experienced larger burnt area for wildfires than previously, which may be due to land management or could be caused the changing meteorological conditions—or a combination.

We assess the chance of any given year exceeding the 1976 and 2022 records, finding values of 1.0% and 5.6 % respectively. As we also show that the model may be underestimating the frequency of extreme values, with no model days exceeding the observed record despite 20× more available data, these estimates are likely conservative. The data presented suggests the model will underestimate the likelihood of extreme FWI days.

Using a large ensemble of initialised climate models we show that fire weather in south‐east England has increased since 1960. We show there has been a 55% increase in high and 240% increase in very high risk days in summertime in south‐east England. As highlighted by Arnell, Freeman, and Gazzard (2021a) there is a need for a fire danger system tailored to the United Kingdom, and careful planning to ensure increasing fire weather risk does not lead to greater impacts.

## Author Contributions

V.T. designed the study and performed the data analysis. N.D. and G.K. supplied the model data. All authors contributed to the interpretation of the result and the writing of the article.

## Conflicts of Interest

The authors declare no conflicts of interest.

## Supporting information




**Figure S1** The implications of the differing variables used calculation of the Fire Weather Index.

**Figure S2** Flow diagram showing stages in the calculation of the Fire Weather Index.

**Figure S3** Relationships between input variables.

**Figure S4** Modelled and observed change in daily fire weather risk.

## Data Availability

ERA5 data was downloaded from the European Centre for Medium‐Range Weather Forecasts (ECMWF), Copernicus Climate Change Service (C3S) at Climate Data Store (CDS; https://cds.climate.copernicus.eu/). The DePreSys4 UK Met Office model data that support the findings of this study are available upon reasonable request from the authors. The code used to generate the figures in this paper and the Supplementary Materials is available from GitHub: https://github.com/vikki‐thompson/wildfire. All data needed to evaluate the conclusions in the paper are present in the paper and/or the Supplementary Materials.
