# Supplementary material for: Detecting Rising Wildfire Risks for South East England
Source: Clim Resil Sustain. 2025 Jan 9;4(1):e70002. doi: 10.1002/cli2.70002 (PMC11737286; doi:10.1002/cli2.70002)
Supplement: Supplementary file 1 — Figure S1 The implications of the differing variables used calculation of the Fire Weather Index. [file CLI2-4-e70002-s001.docx]

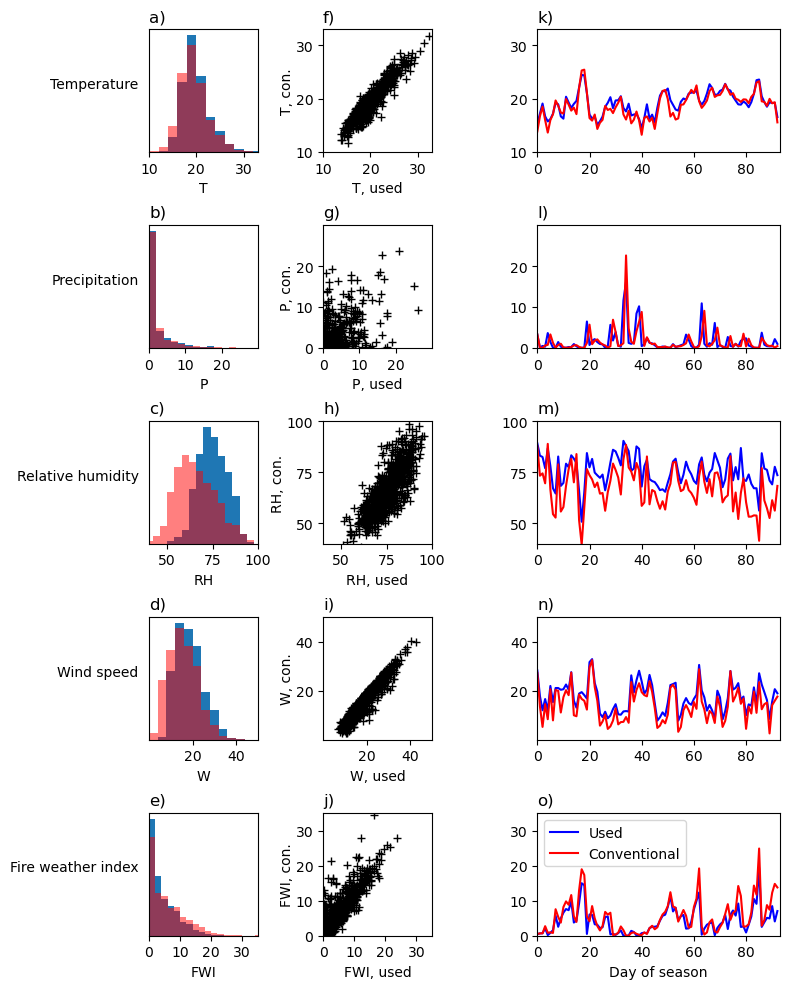


Fig.S1 **The implications of the differing variables used calculation of the Fire Weather Index.** Due to availability of model output the study uses different input variables to those conventionally used to calculate the Fire Weather Index. The conventional inputs are noon values for temperature, relative humidity, and wind, and the total precipitation for the preceding 24 hours (noon to noon). We use maximum daily temperature, daily mean relative humidity, wind speed, and total daily precipitation. **(a-e)** show the ERA5 reanalysis distributions of 2000 to 2010 June-July-August temperature, precipitation, relative humidity, and wind speed, red is the conventional input and blue the input used in this study. **(f-j)** conventional input plotted against the input we used. **(k-o)** timeseries of the daily data for June-July-August of the year 2000 for each variable (red is the conventional, and blue is the input we used).


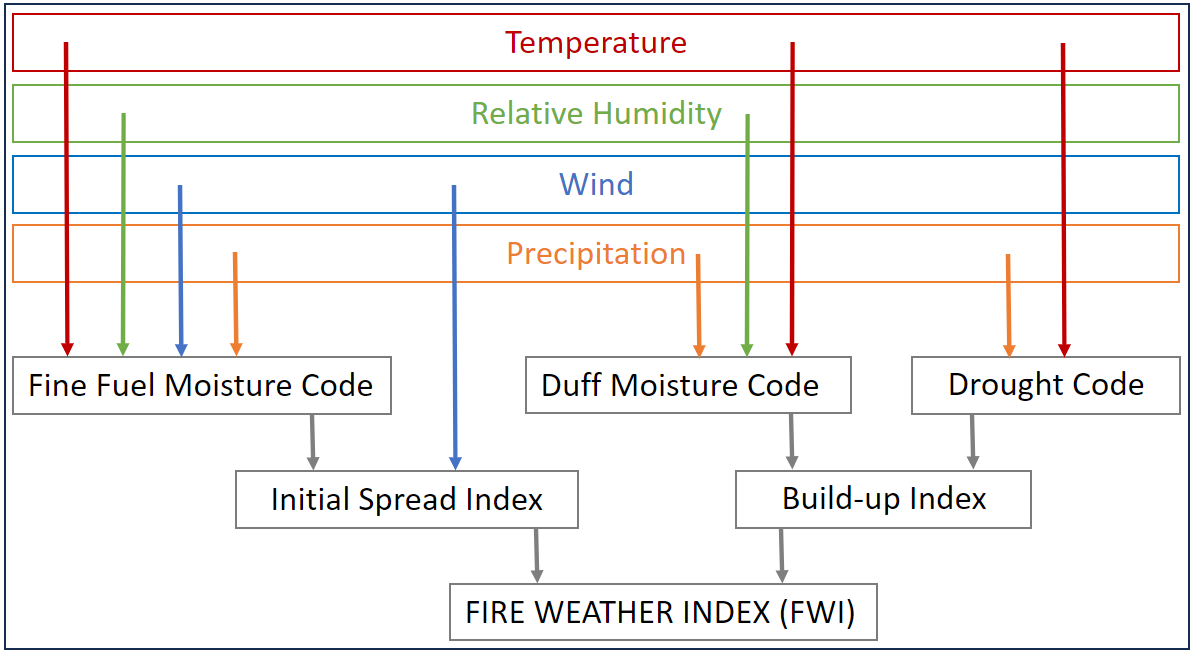


Fig.S2 **Flow diagram showing stages in the calculation of the Fire Weather Index.** A breakdown of how the four climatic variables are used in the calculation of the various stages of the Fire Weather Index calculation.


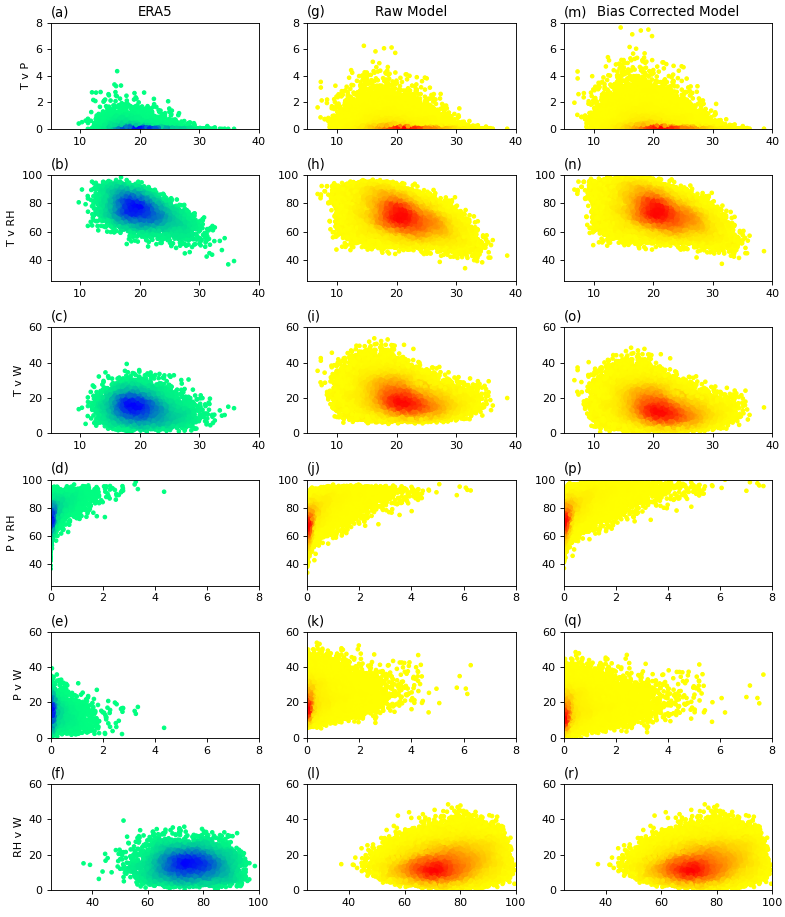


Fig.S3 **Relationships between input variables.** Density plots of each pair of variables for ERA5 (left column), the raw model data (centre column), and the bias corrected model data (right column). For ERA5 dark blue indicates higher data density, and for the model data red indicates higher density. Each row is a different pair of variables: T = temperature (°C), P = precipitation (mm), RH = relative humidity (%), and W = wind speed (m/s). Axes limits are consistent for each variable.


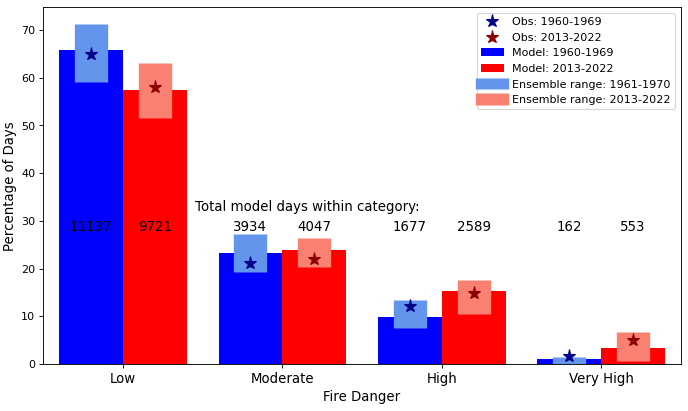


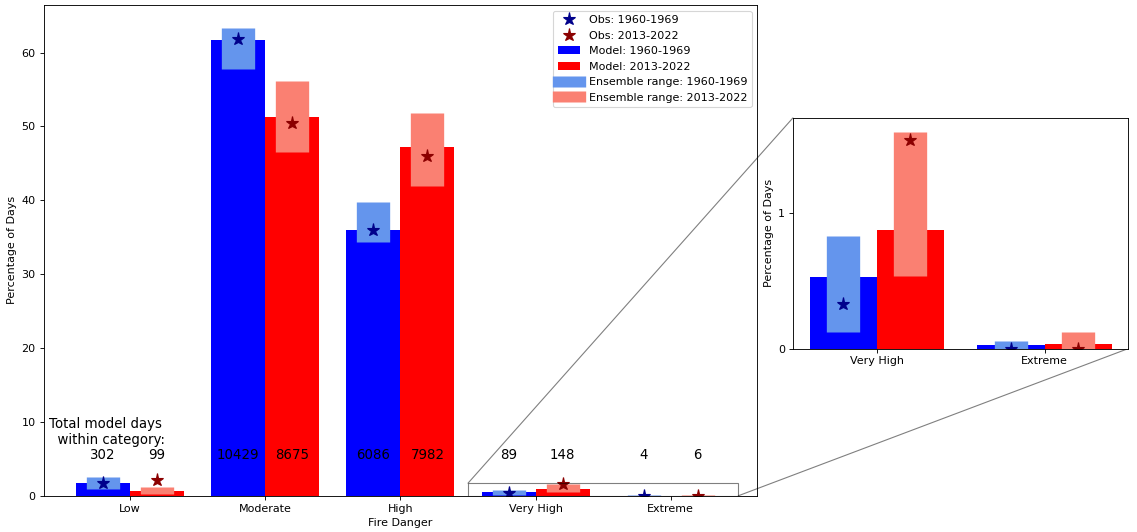


Fig. S4 **Modelled and observed change in daily fire weather risk.** Showing the percentage of days within each fire danger category in the past (1961-1970) in blue, and present (2013-2022) in red for summertime days, using the large ensemble of initialised climate model data. The absolute values for number of days is shown for the full ensemble, uncertainty is represented by the ensemble range, and the observed values are shown as stars.
